# Editorial: From Organizational Welfare to Business Success: Higher Performance in Healthy Organizational Environments

**DOI:** 10.3389/fpsyg.2017.00720

**Published:** 2017-05-23

**Authors:** Gabriele Giorgi, Mindy Shoss, Annamaria Di Fabio

**Affiliations:** ^1^Department of Human Science, Psychology Section, European University of RomeRome, Italy; ^2^Department of Psychology, University of Central FloridaOrlando, FL, USA; ^3^Department of Education and Psychology, Psychology Section, University of FlorenceFirenze, Italy

**Keywords:** organizational psychology, performance, healthy organization, business, work-related stress, occupational health

## Introduction

This special issue provides insight into the link between employee health and productivity/performance, with a focus on how individuals, groups, or organizations can intervene in this relationship to improve both well-being and performance-related outcomes. Given the continuous changes that organizations and employees face, such as the aging workforce and continued economic turbulence, it is not surprising that studies are increasingly finding that employee health is related to job conditions. The papers in this special issue emphasize that organizations make a critical difference when it comes to employees' health and well-being. In turn, healthy employees help their organizations to flourish. Such findings are in line with the recent emphasis by both the International Labour Organization (ILO) and the United Nations (UN) on the importance of work for individual well-being and the importance of individual well-being for productive and sustainable economic growth (see e.g., ILO, 1985; World Health Organisation, 2007; UN, 2015).

The 21 papers accepted in this special issue are predominately empirical contributions, from authors from different disciplinary sectors (Organizational Psychology, Management, Occupational Medicine, and Engineering) and several different regions (Southern-Europe, Northern-Europe, Eastern-Europe, United States, and Australia). As a result, they offer diverse perspectives on the issue of employee health and performance, and help to promote an international and interdisciplinary approach to research that will enable the progress of science in the business-health field.

Taken together, all of the papers in this special issue emphasize three important points. First, issues of employee health and well-being are especially pressing in light of increasing turbulence and instability in business and in the global economy. Second, employee health and well-being are crucial for organizational success. Poor working conditions and environments (including relational environments) ultimately harm performance. In contrast, organizations that foster healthy environments promote both employee and organizational success. Third, efforts to address employee health must be multi-pronged and involve individual, unit, and organizational strategies.

## Overview of articles in this research topic

The 21 papers published in the special issue addressed these issues in a variety of ways.

Two studies examined the *impact of personality on well-being, yielding insights into which workers are most susceptible to poor well-being at work*. First, the article by Ceschi et al. was conducted in a sample of 208 private service sector Italian employees. Their findings underscored the importance of personality traits in the relationship between health impairment process and the emergence of counterproductive work behavior. Interestingly, honesty-humility seemed to have negative consequences for workers' burnout. In the second article, Di Fabio and Kenny adopted a primary prevention perspective in their examination of emotional intelligence and well-being among Italian high school students. They found that trait emotional intelligence impacts well-being even when controlling for personality and fluid intelligence, and their paper offers insights for developing future workers.

Four articles examined *causes and consequences of stressors at work*. First, Giorgi et al. presented a new theoretical and empirical model investigating multiple fears in workplace among 265 Italian expatriate workers. Using a structural equation model design, this innovative model explored the relationships of different fears, fear of expatriation, fear of the economic crisis, and dangerous working conditions into an composite framework. Second, in a large study of Italian workers, Di Marco et al. found that perceptions of a discriminatory work environment threaten employee health, and that these effects are partially mediated by job satisfaction. They suggest practical implications for organizations. Third, Mucci et al. examined the impact of anxiety and work-related stress on hypertension among students training to be healthcare professionals. They found evidence linking psychological stressors to students' blood pressure and cardiovascular health. These findings demonstrate that even among a group of relatively young individuals, work-related stressors are related to cardiovascular health. Fourth, Spanouli and Hofmans pointed out that employees might increasingly feel obligated to perform citizenship behaviors at work. Using an experience sampling design, they asked employees to report on their elicited and discretionary organizational citizenship behavior and counterproductive work behavior twice a day for 10 days. Their results suggest that these strategies are likely to backfire as individuals appear to use counterproductive work behavior to compensate for elicited organizational citizenship behavior. Each of these studies suggests areas to intervene to reduce or eliminate threats to health and well-being at work.

Relatedly, the majority of studies featured in this special issue explored *individual, unit, and/or organizational strategies to improve health and well-being*. The article by Dijkstra and Homan, conducted in a heterogeneous sample of 543 Dutch employees, found that engaged coping strategies such as active confronting and reassuring thoughts were associated with more sense of control and therefore to psychological well-being. This publication suggests that the importance of the concept of coping is nowadays still fundamental. Leon-Perez et al. took a multilevel approach to understanding how intragroup conflict, at the unit level, and psychological capital, at the individual level, impacts employees' burnout and quality of service. In a sample of employees working in a vehicle safety and emissions inspection company in Spain, they found that individual psychological capital impacted both burnout and quality of service. At the unit level, they found that intragroup conflict also predicted burnout, and that units' conflict management climate can buffer the harmful impact of conflict on quality of service. Di Fabio et al. argued that relational incivility is crucial for positive outcomes when organizations undergo change. Their study, conducted with a sample of 261 Italian workers, found that relational civility positively related not only to well-being outcomes, but also to acceptance of change.

The article by Pignata et al. using the Job Demands-Resources theoretical model of stress investigated the antecedents of perceived procedural justice in a two-wave longitudinal sample of 945 employees from 13 Australian universities. The article findings stimulate the development of more fair and transparent processes in the academic setting in order to increase justice perceptions. Relatedly, the article by Giorgi et al. determined the advantage of using recursive partitioning in the prediction of perceived organizational support in a sample of more than 6,000 Italian bankers. A regression tree model was estimated utilizing the tree function party package in R. The emerged results seem strategic both for research and practice. The article by Guglielmi et al. proposes an interesting model of engagement among older workers. They surveyed older workers in Northern Italy and found evidence of a gain cycle wherein job satisfaction increases work engagement, which in turn impacts subsequent job satisfaction. Job demands and age play a role in the strength of this gain cycle.

The article by Page and Nilsson used an innovative approach to improve employees' well-being and organizational behavior. Active commuting using electrically assisted bikes (e-bikes) resulted in more productive and healthy organizational behavior outcomes compared with passive commuters. Pignata et al. shed light on the important issue of awareness of organizational stress interventions. In a two-wave longitudinal study of employees from 13 Australian universities, they find that awareness of stress interventions plays an important role in impacting satisfaction, trust, and for non-academic employees, commitment, and justice perceptions. These results varied depending on organizational tenure, suggesting that organizations might need to promote interventions differently depending on tenure. Finally, Cortini et al. examine the relationship between learning climate and job performance. In a sample of health professionals gathered from a public hospital in Italy, they found evidence that a learning climate promotes job performance by virtue of impacting psychological strain.

Additionally, many of the articles in the special issue offer *tools for employees and organizations to monitor and improve health and well-being*. First, the article by Petrović et al. examined the cut off scores of the most scientifically recognized instrument of workplace bullying measurement: the Negative Acts Questionnaire Revised (NAQ-R). In the cultural and economic context of Serbia, this article is an important reference for researchers and practitioners who wish to use this questionnaire. Second, Di Fabio et al. evaluated the psychometric properties of a new multi-dimensional scale for entrepreneurship, leadership, and professionalism. Entrepreneurship, leadership, and professionalism are increasingly important in today's ever-changing and uncertain business environment. This tool is likely to aid both research and practice. The third tool is addressed in the article by Di Fabio. In particular, this article examined the psychometric properties of the Positive Relational Management Scale (PRMS). This scale demonstrated good psychometric properties, and can serve as another useful tool for assessing and promoting positive experiences at work. Third, Loera et al. offer evidence for the validity and measurement invariance of the User-Initiated Support Scale, which assesses social support from users (e.g., students, patients). This tool assesses a crucial, yet often ignored, source of social support at work. Fourth, Pedrazza et al. reported on the development of a scale to assess physician's dissatisfaction and work-related stress. They explore various dimensions of stressful experiences among physicians and highlight role uncertainty and loss of social esteem as particularly stressful factors. Finally, in a two-year action research project taking place in the operating rooms of six hospitals in Northern Italy, Bruno and Bracco developed an innovative methodology for noticing and monitoring critical threats to safety and well-being. This tool serves as an excellent resource for monitoring and addressing safety and well-being at work in a proactive, team-oriented manner.

## Conclusions

Overall, the papers in this special issue report findings from a cumulative sample of nearly 19,000 workers and perspectives from 68 authors. They suggest that performance cannot be successfully achieved at the cost of health and well-being, and provide various perspectives and tools to guide future research and practice.

We conclude with a motto of the Business@health laboratory of the European University of Rome (www.uerbusinesshealth.com) “***Business doesn't exist without workers' health & workers' health is business***.” We hope that this special issue will inspire researchers and practitioners to expand their horizons to integrate occupational health and safety protection as part of activities designed to promote well-being, performance, and productivity (e.g., Danna and Griffin, 1999; Lerner et al., 2013; Di Fabio and Kenny, 2016).

## Author contributions

All authors listed, have made substantial, direct, and intellectual contribution to the work, and approved it for publication.

### Conflict of interest statement

The authors declare that the research was conducted in the absence of any commercial or financial relationships that could be construed as a potential conflict of interest.
